# Inflexible Youngsters: Psychological and Psychopathological Correlates of the Avoidance and Fusion Questionnaire for Youths in Nonclinical Dutch Adolescents

**DOI:** 10.1007/s12671-017-0714-1

**Published:** 2017-03-27

**Authors:** Peter Muris, Cor Meesters, Anke Herings, Marieke Jansen, Chris Vossen, Pina Kersten

**Affiliations:** Department of Clinical Psychological Science, Maastricht University, P.O. Box 616, 6200 MD Maastricht, The Netherlands

**Keywords:** Psychological inflexibility, Avoidance and Fusion Questionnaire for Youth (AYQ-Y), Internalizing and externalizing, Adolescents

## Abstract

The present study examined psychological and psychopathological correlates of psychological inflexibility as measured by the Avoidance and Fusion Questionnaire for Youth (AFQ-Y) in two independent samples of nonclinical Dutch adolescents aged between 12 and 18 years (*N*s being 184 and 157). Participants completed a survey containing the AFQ-Y and scales assessing mindfulness, thought suppression, self-compassion, self-worth, self-efficacy, and internalizing/externalizing symptoms. In both samples, the AFQ-Y was found to be a reliable measure of psychological inflexibility that correlated in a theoretically meaningful way with other psychological constructs. Most importantly, AFQ-Y scores correlated positively with internalizing and externalizing symptoms, and in most cases, these associations remained significant when controlling for other measures. These findings suggest that psychological inflexibility is an important factor in youth psychopathology that needs to be further investigated in future research.

## Introduction

Psychological flexibility can be described as the ability to maintain present-moment awareness, in which the person is capable—depending on what the situation affords—of changing or maintaining actions to pursue valued goals and interests (Hayes et al. 1999). While psychological flexibility is assumed to promote people’s well-being, it appears also true that a lack of this quality, which can be labeled as psychological inflexibility, is associated with a heightened risk for developing mental health problems (Kashdan and Rottenberg 2010). Psychological inflexibility is thought to be produced by two processes: cognitive fusion and experiential avoidance. Cognitive fusion is the process by which thoughts about an event become merged with the actual event. In other words, thoughts are taken as so truly real that, instead of considering them as mental events that do not necessarily require action, the person is fully dominated by them (Luoma and Hayes 2003; Solé et al. 2016). Experiential avoidance refers to the tendency to avert unwanted thoughts and feelings, resulting in deliberate efforts to change their content and frequency (Hayes et al. 1999; Valdivia-Salas et al. in press).

On first sight, cognitive fusion and experiential avoidance seem quite different phenomena, but a simple example such as “I look stupid (i.e., cognitive fusion), so I better not go to the party” (experiential avoidance) makes clear that both components are often intimately related, thereby equally contributing to the single theoretical construct of psychological inflexibility. It is evident that cognitive fusion and experiential avoidance are prominent in emotional disorders such as depression and anxiety disorders, which are both characterized by pervasive negative mood and intrusive thinking as well as inflexible avoidant behavioral and cognitive responses (Kashdan and Rottenberg 2010). Yet, even in other types of psychopathology such as somatization (e.g., Wicksell et al. 2008) and behavioral disorders (Livheim et al. 2016), these processes of psychological inflexibility seem to be present.

Adolescence is characterized by marked physical, cognitive, and social changes, and therefore, scholars tend to define it as a period of “storm and stress” (e.g., Jensen Arnett 1999). Episodes of anxiety, sadness, anger, and aggression commonly occur in young people, and a substantial minority of them display emotional and behavioral dysregulations (e.g., Wills et al. 2016) and may be prone to develop a psychiatric disorder (Costello et al. 2003). The presence of psychological flexibility may help the adolescent to deal effectively with the challenges posed by this developmental stage (Wenar and Kerig 2000). In contrast, when psychological inflexibility prevails, the young person may frequently engage in considerable attempts to control emotions, thoughts, and behaviors by excessively and rigidly applying certain regulatory strategies, which is a hallmark of many forms of psychopathology (Kashdan and Rottenberg 2010).

To assess individual differences in psychological inflexibility in young people, Greco et al. (2008) developed the Avoidance and Fusion Questionnaire for Youth (AFQ-Y). This self-report scale consists of 17 items measuring the processes of cognitive fusion and experiential avoidance, which jointly constitute the construct of psychological inflexibility. The total score of the AFQ-Y has proven to be reliable and correlates in a theoretically meaningful way with related psychological constructs to support its convergent validity. That is, positive correlations have been documented with thought suppression (Wegner 1989), which is a cognitive manifestation of the process of experiential avoidance, whereas negative correlations have been found with mindfulness (Bishop et al. 2004), which can be regarded as antithetical to cognitive fusion (see Greco et al. 2008). In addition, the AFQ-Y has also been shown to correlate positively with measures of psychopathological symptoms (Greco et al. 2008; Feinstein et al. 2011; Fergus et al. 2012; Livheim et al. 2016; Paulus et al. 2016; Simon and Verboon 2016; Valdivia-Salas et al. in press; Venta et al. 2012), although it should be noted that most of this research focused on the relation between psychological inflexibility and internalizing (emotional) symptoms.

It can be hypothesized that psychological inflexibility is related to self-related constructs. A case in point is self-compassion, which can be defined as the tendency to be caring, warm, and understanding towards oneself when faced with personal shortcomings, inadequacies, or failures (Neff 2003a). According to Hayes et al. (1999), lack of self-compassion intersects with psychological inflexibility and in its wake psychopathology, and there is indeed emerging empirical evidence to support this notion (Marshall and Brockman 2016). Another example is self-worth, which refers to the general feeling that one is good as a person and deserves to be treated with respect (Rosenberg 1979). This characteristic appears to be absent in psychologically inflexible persons who are often fused with negative thinking about themselves and at the same time are experientially avoidant about their negative attributes (Al-Jabari 2012). The third and final self-related construct that may be related to psychological inflexibility is self-efficacy. Self-efficacy pertains to the belief in one’s ability to plan and execute required behaviors in prospective situations (Bandura 1977). This concept is strongly related to self-control, which is considered by some as a prerequisite (Kashdan and Rottenberg 2010) and by others as a consequence (Ruiz 2014) of psychological flexibility. Either way, it can be expected that psychological flexibility is positively related to self-efficacy, and consequently that inflexibility is associated with low levels of this self-related construct.

As noted earlier, previous research has been mainly concerned with internalizing symptoms such as anxiety, depression, and somatic complaints (Feinstein et al. 2011; Fergus et al. 2012; Greco et al. 2008; Livheim et al. 2016; Paulus et al. 2016; Simon and Verboon 2016; Valdivia-Salas et al. in press; Venta et al. 2012). One of these studies (Greco et al. 2008) included an assessment instrument that measured internalizing symptoms as well as symptoms of hyperactivity and oppositional behavior, but failed to analyze the relations between psychological inflexibility and internalizing and externalizing symptoms separately. Another investigation by Livheim et al. (2016) employed the Beck Youth Inventories (Beck et al. 2005) and did conduct separate analyses for anxiety/depression and anger/disruptive behavior. The results showed that psychological inflexibility is not only positively related to internalizing symptoms but also (although to a lesser extent) to externalizing symptoms. This fits well with the clinical picture of youths with externalizing problems. These youngsters often display hostile, angry, resentful, and aggressive thoughts (e.g., Schniering and Rapee 2004)—which can be seen as a manifestation of cognitive fusion, and tend to be avoidant about their own share in negative events and problems occurring in life (e.g., Powell et al. 1997)—which can be regarded as a variant of experiential avoidance.

The purpose of the present study was to further examine the construct of psychological inflexibility in youths. Two independent samples of adolescents completed the AFQ-Y as well as scales measuring related psychological constructs and psychopathological symptoms. The research not only investigated a number of basic psychometric properties of the AFQ-Y but also addressed three topics that were not or only partly addressed in previous studies. First, the relations between psychological inflexibility and a number of self-related constructs (i.e., self-compassion, self-worth, and self-efficacy) were explored. Second, the relations between psychological inflexibility and internalizing as well as externalizing symptoms were examined. Third, given the presumed central role of psychological inflexibility in mental health problems (Kashdan and Rottenberg 2010) and because the present study included a large number of related variables, we were also interested in examining the unique contribution of psychological inflexibility to youths’ internalizing and externalizing symptoms. More precisely, we wanted to explore the incremental validity of the AFQ-Y in predicting psychopathological symptoms while controlling for other variables that may be relevant in this regard (i.e., thought suppression, mindfulness, self-compassion, self-worth, and self-efficacy). Such analysis is important in order to establish the relative importance of psychological inflexibility among other factors related to mental health problems (Woodruff et al. 2014).

## Method

### Participants

Sample 1 was recruited at Zwijsen College in Veghel. One hundred eighty-four adolescents (84 boys and 100 girls) participated and completed the full survey (response rate 78.6%). Their mean age was 13.64 years (*SD* = 1.04, range 12–16 years). Youths either followed higher general continued education (71.7%) or pre-university secondary education (28.3%). Sample 2 consisted of pupils from Bouwens Van der Boije College in Panningen and Rombouts College in Brunssum. Here, 157 adolescents (50 boys, 107 girls) with a mean age of 15.33 years (*SD* = 1.31, range 12–18 years) took part (response rate 28.9%). In this sample, three educational levels were included: lower vocational education (19.7%), higher general continued education (58.0%), and pre-university secondary education (22.3%).

### Procedure

Two independent samples were recruited via regular high schools in the southern part of The Netherlands. First, the schools were approached to check their willingness to participate in this research. In schools that decided to take part in the study, informed consent letters were distributed to pupils and parents. In case of a positive decision regarding participation (response rates are given below), adolescents completed a booklet containing various questionnaires. This assessment took place at school, during regular classes. Research assistants were always present to provide the young participants with a general instruction on how to complete the survey, to clarify items, and to ensure confidential and independent responding.

### Measures

#### Psychological Inflexibility

The *AFQ-Y* (Greco et al. 2008) is a 17-item self-report scale for measuring psychological inflexibility in children and adolescents. Items represent the processes of cognitive fusion (e.g., “The bad things that I think about myself must be true”) and experiential avoidance (e.g., “I push away thoughts and feelings that I don’t like”) and are rated on a five-point scale with 1 = not at all true and 5 = very true. Given the strong relation between cognitive fusion and experiential avoidance, the developers did not create subscales for these two processes but rather treat the AFQ-Y as a unidimensional measure (Greco et al. 2008). A total score is obtained by summing the ratings on all items, with a higher score indicating a higher level of psychological inflexibility. As already noted in the “Introduction,” there is good evidence for the reliability and validity of the AFQ-Y.

#### Mindfulness and Thought Suppression

The *Child and Adolescent Mindfulness Measure* (CAMM; Greco et al. 2011) consists of ten items that intend to assess present-moment awareness and nonjudgmental, nonavoidant responses to thoughts and feelings in youths. Items are scored on a five-point rating scale (0 = never true, 4 = always true) and are all negatively formulated (e.g., “At school, I walk from class to class without noticing what I’m doing”), so that they need to be reversed before combining them to a total score for which higher scores reflect higher levels of mindfulness. Psychometric evaluations have demonstrated that the CAMM is a reliable scale that correlates positively with measures of quality of life, self-regulation, social skills, and academic performance (De Bruin et al. 2014; Greco et al. 2011) and negatively with indices of stress and psychopathology (De Bruin et al. 2014; Greco et al. 2011; Kuby et al. 2015).

The *White Bear Suppression Inventory* (WBSI; Wegner and Zanakos 1994) is a 15-item questionnaire measuring unwanted intrusive thoughts as well as the tendency to suppress such thoughts. The scale asks respondents to indicate on a five-point scale the extent to which they agree (1 = strongly disagree; 5 = strongly agree) with statements such as “There are things I prefer not to think about,” “I have thoughts I cannot stop,” and “There are thoughts that keep jumping into my head.” Responses are summed to yield a total score that ranges from 15 to 75. Higher scores indicate higher levels of intrusive thinking and a stronger tendency to suppress unwanted thoughts. The reliability and validity of the WBSI is satisfactory (Wegner and Zanakos 1994; Muris et al. 1996), and this has also been shown to be the case in children and adolescents (Vincken et al. 2012).

### Self-Related Constructs

The *Shortened Self-Compassion Scale for Adolescents* (S-SCS-A; Muris et al. 2016), which is an abbreviated, age-downward version of the Self-Compassion Scale (SCS; Neff 2003b), contains nine items representing the three key components of self-compassion, namely self-kindness (e.g., “When I feel sad, I try to be tender to myself”), common humanity (e.g., “When I have problems, I remind myself that everybody has difficulties from time to time”), and mindfulness (e.g., “When I am feeling down, I am still able to think about positive things”). Items are rated on five-point scales (1 = never, 5 = always) and then summed to produce a total score, for which higher scores are indicative of higher levels of self-compassion. Preliminary evidence for the reliability and validity of the S-SCS-A has been found (Muris et al. 2016). That is, the Cronbach’s alpha of the total scale is well above 0.80 and scores are found to relate substantially with the Short Form SCS for adults (SF-SCS; Raes et al. 2011) as well as with other self-related constructs (e.g., self-esteem, self-efficacy).

Self-worth was assessed by means of a subscale of the *Self-Perception Profile for Children* (SPPC; Harter 1985). This subscale contains six items that each consist of two opposite descriptions, e.g., “Some kids are often unhappy with themselves” but “Other kids are pretty pleased with themselves.” Participants first choose the description that best fits and then indicate whether the description is *somewhat true* or *very true* for them. Accordingly, each item is scored on a four-point scale. A total score can be computed, with a higher score reflecting a more positive view of oneself. Psychometric evaluations of the SPPC have indicated that this scale provides a reliable and valid index of global self-worth in children and adolescents (e.g., Harter 1985; Muris et al. 2003)

The *Self-Efficacy Questionnaire for Children* (Muris 2001) is composed of 24 items assessing perceptions of self-efficacy in three domains: (1) social self-efficacy (eight items) which has to do with the perceived capability for dealing in an effective way with other people (e.g., “How well can you become friends with other children?”); (2) academic self-efficacy (eight items) which is concerned with the perceived capability to manage one’s academic affairs (e.g., “How well can you study when there are other interesting things to do?”); and (3) emotional self-efficacy (eight items) which pertains to the perceived capability of coping with negative emotions (e.g., “How well can you control your feelings?”). Each item has to be scored on a five-point scale with 1 = *not at all* and 5 = *very well*. A total self-efficacy score can be computed by summing all items. Research has yielded support for the reliability and validity of the SEQ-C (Muris 2001; Suldo and Shaffer 2007).

### Psychopathological Symptoms

The *Children’s Somatization Inventory-Short Form* (CSI-SF; Walker and Garber 2001; see Walker and Lambert 2011) measures to what extent (0 = not at all; 4 = very much) young people are bothered by eight common physical-somatic symptoms (i.e., headache, stomach pain, weakness, low back pain, faintness, arm/leg pain, heart beating too fast, and nausea) during the past 2 weeks. A total somatization score can be obtained by summing all items. The CSI has good psychometric qualities (e.g., Meesters et al. 2003; Walker et al. 2009), and this seems also true for the shortened version (Walker and Lambert 2011).

The *Youth Self-Report* (YSR; Achenbach and Rescorla 2001; Verhulst et al. 1997) is a widely used questionnaire for measuring psychopathological symptoms in youths aged 11 years and older. The full instrument contains 112 problem items for which the young informant has to rate to what extent they are applicable (0 = not true, 1 = somewhat true, and 2 = very true). Items can be combined into DSM-oriented scales, of which the following four were used in this study: affective problems (13 items, e.g., “There are few things that I enjoy”), anxiety problems (6 items, e.g., “I am too scared or anxious”), and oppositional and conduct problems (which were combined because of the low frequency of these symptoms; 21 items, e.g., “I am disobedient at home”, “I fight a lot”). There is abundant research to support the reliability and validity of the YSR (Achenbach 2009).

The *Youth Anxiety Measure for DSM-5* (YAM-5; Muris et al. 2017) is a recently developed questionnaire for assessing symptoms of anxiety disorders as listed in DSM-5 (American Psychiatric Association [APA] 2013). The first part of the YAM-5 addresses symptoms of the major anxiety disorders, while the second part is concerned with symptoms of phobias. In the present study, only the first part was used, which measures symptoms of separation anxiety disorder (e.g., “I get frightened if my parents leave the house without me”), selective mutism (e.g., “At school I don’t speak to the teacher at all”), social anxiety disorder (e.g., “I find it scary to eat or drink if other people are looking at me”), panic disorder (e.g., “I suffer from anxiety or panic attacks”), and generalized anxiety disorder (e.g., “I worry about a lot of things”). Children are asked to rate the frequency of each of the 28 items using a four-point Likert-type scale with 0 = never, 1 = sometimes, 2 = most of the time, and 3 = always. Ratings are summed to yield subscale scores as well as a total score, which was used in the present study. There is emerging support for the psychometric qualities of the YAM-5 (Garcia-Lopez et al. 2017; Muris et al. 2017).

The shortened version of the Revised Child Anxiety and Depression Scale (RCADS; Chorpita et al. 2000; Muris et al. 2002) consists of 25 items that not only assess symptoms of DSM-IV (APA 1994) defined anxiety disorders (i.e., social phobia, generalized anxiety disorder, separation anxiety disorder, and panic disorder) but also symptoms of major depressive disorder. Items (e.g., “I am scared when I have to take a test”, “I feel sad and empty”) have to be scored on a 4-point scale with 0 = never, 1 = sometimes, 2 = often, and 3 = always, and can be combined into a total anxiety and a total depression score. The reliability and validity of the RCADS are good, and this is also true for the shortened version of the scale (Muris et al. 2002).

The *Child Rating scale for Aggression* (CRA) is a self-report version of the Teacher Rating scale for Aggression (TRA; Brown et al. 1996), which consists of 21 items referring to aggressive feelings and behaviors of children and adolescents: 6 items represent reactive aggression (e.g., “I get angry for no reason”), 10 items reflect proactive aggression (e.g., “I am mean”), whereas 5 items cannot be classified in these two categories. Each item has to be rated on a five-point scale ranging from 1 = never to 5 = almost always. Research has demonstrated that the CRA is reliable in terms of internal consistency (with Cronbach’s alphas in the 0.80 range; e.g., Meesters et al. 2007), and correlates significantly with other measures of self-reported disruptive behavior problems such as the externalizing scale of the YSR (Vincken 2003).

Participants of sample 1 completed the AFQ-Y, CAMM, WBSI, S-SCS-A, SPPC, CSI, and YSR, while those of sample 2 filled out the AFQ-Y, CAMM, WBSI, S-SCS-A, SPPC, SEQ-C, YAM-5, RCADS, and CRA. The AFQ-Y, being the central measure in this research, was always administered first. The other questionnaires were taken in two reversed orders.

### Data Analyses

The Statistical Package of Social Sciences (SPSS) was employed to compute descriptive statistics and reliability coefficients (Cronbach’s alphas) for various questionnaires. Correlational analyses were then conducted to explore the relations among psychological inflexibility as indexed by the AFQ-Y and other constructs. To compare the strength of the correlations between the AFQ-Y and internalizing and externalizing symptoms, we conducted tests for comparing correlated correlation coefficients (Meng et al. 1992). The unique contribution of psychological inflexibility to youths’ internalizing and externalizing symptoms was examined by means of hierarchical regression analyses. In these analyses, other psychological constructs were entered on step 1, while psychological inflexibility (AFQ-Y) was added to the equation on step 2 to investigate its unique predictive value of psychopathological symptoms.

## Results

### Sample 1

#### General Findings

The AFQ-Y was found to be a reliable scale with a Cronbach’s alpha of 0.80, and this appeared also true for most of the other scales (with Cronbach’s alphas generally well above 0.70; Table 1). The only exception was the oppositional/conduct problems scale of the YSR, which had an alpha of 0.69. This was primarily due to a number of items (on truancy, fire setting, and stealing) that were rarely endorsed by the participants. In addition, *t* tests revealed statistically significant gender differences for SPPC self-worth [*t*(182) = 2.91, *p* < .01], CSI somatization [*t*(180.81) = 4.08, *p* < .001], YSR anxiety problems [*t*(181.57) = 3.24, *p* < .01], YSR affective problems [*t*(181.96) = 4.11, *p* < .001], and YSR oppositional/conduct problems [*t*(182) = 2.30, *p* < .05]. As can be seen in Table 1, girls displayed higher levels of somatization, anxiety, and depression, whereas boys scored higher on self-worth and oppositional/conduct problems. Given these differences, we decided to control for gender in further analyses. It should be noted that when not controlling for gender, highly similar results were documented.

**Table 1:** Descriptive statistics (means, standard deviations, gender differences, and internal consistency coefficients) for various measures obtained in sample 1

	Total sample (*N* = 184)	Boys (*n* = 84)	Girls (*n* = 100)	Cronbach’s α
AFQ-Y psychological inflexibility	18.20 (8.68)	16.97 (7.37)_a_	19.24 (9.56)_a_	0.80
CAMM mindfulness	26.67 (6.07)	26.96 (6.26)_a_	26.43 (5.92)_a_	0.76
WBSI thought suppression	42.70 (13.00)	40.93 (12.88)_a_	44.18 (12.97)_a_	0.91
S-SCS-A self-compassion	26.35 (6.67)	26.27 (7.11)_a_	26.42 (6.32)_a_	0.84
SPPC self-worth	14.33 (5.78)	15.65 (4.96)_a_	13.22 (6.19)_b_	0.81
CSI somatization	7.40 (4.96)	5.87 (4.08)_a_	8.69 (5.28)_b_	0.72
YSR anxiety problems	2.86 (2.16)	2.19 (1.85)_a_	3.43 (2.24)_b_	0.71
YSR affective problems	4.68 (3.55)	3.80 (3.03)_a_	5.43 (3.79)_b_	0.74
YSR oppositional/conduct problems	5.03 (3.14)	5.61 (3.46)_a_	4.55 (2.78)_b_	0.69

Means not sharing similar subscripts differ at *p* < .05

*AFQ-Y* Avoidance and Fusion Questionnaire for Youth, *CAMM* Child and Adolescent Mindfulness Measure, *WBSI* White Bear Suppression Inventory, *S-SCS-A* Shortened Self-Compassion Scale for Adolescents, *SPPC* Self-Perception Profile for Children, *CSI* Children’s Somatization Inventory, *YSR* Youth Self-Report

#### Relations Between Psychological Flexibility and Other Psychological Constructs/Symptoms

Table 2 shows the correlations (corrected for gender) between psychological inflexibility and psychological constructs/symptoms. As expected, the AFQ-Y correlated positively with the WBSI (*r* = .63) and negatively with the CAMM (*r* = −.55), S-SCS-A (*r* = −.23), and SPPC (*r* = −.41), which means that higher levels of psychological inflexibility were associated with higher levels of thought suppression and lower levels of mindfulness, self-compassion, and self-worth. With the CSI and YSR scales, positive correlations were found, which implies that higher levels of psychological inflexibility were accompanied by higher levels of symptoms. The most robust correlation was found between the AFQ-Y and YSR affective problems (*r* = .54), and tests for comparing correlated correlation coefficients revealed that this correlation was significantly stronger than the correlations between the AFQ-Y and YSR oppositional/conduct problems (*r* = .31; *z* = 3.13, *p* < .01) and CSI somatization (*r* = .38; *z* = 2.41, *p* < .05).

**Table 2:** Correlations (corrected for gender) among AFQ-Y and other measures (sample 1)

	1	2	3	4	5	6	7	8
1. AFQ-Y psychological inflexibility								
2. CAMM mindfulness	−.55**							
3. WBSI thought suppression	.63**	−.66**						
4. S-SCS-A self-compassion	−.23*	.07	−.15					
5. SPPC self-worth	−.41**	.29**	−.29**	.35**				
6. CSI somatization	.38**	−.37**	.39**	−.18*	−.27**			
7. YSR anxiety problems	.45**	−.37**	.41**	−.19*	−.38**	.40**		
8. YSR affective problems	.54**	−.44**	.44**	−.30**	−.40**	.45**	.48**	
9. YSR oppositional/conduct problems	.31**	−.27**	.21*	−.19*	−.25*	.26**	.28**	.35**

*N* = 184

*AFQ-Y* Avoidance and Fusion Questionnaire for Youth, *CAMM* Child and Adolescent Mindfulness Measure, *WBSI* White Bear Suppression Inventory, *S-SCS-A* Shortened Self-Compassion Scale for Adolescents, *SPPC* Self-Perception Profile for Children, *CSI* Children’s Somatization Inventory, *YSR* Youth Self-Report

*p < .05, **p < .001

There were also significant correlations among the other psychological constructs (Table 2). That is, WBSI thought suppression correlated negatively with CAMM mindfulness (*r* = −.66, *p* < .001), and SPPC self-worth (*r* = −.29, *p* < .001), while SPPC self-worth correlated positively with S-SCS-A self-compassion (*r* = .35, *p* < .001) and CAMM mindfulness (*r* = .29, *p* < .001).

#### Unique Contribution of Psychological Inflexibility to Symptoms

Hierarchical regression analyses were carried out in which CSI and various YSR scales were the dependent variables. Predictors were mindfulness (CAMM), thought suppression (WBSI), self-compassion (S-SCS-A), and self-worth (SPPC), which were entered on step 1, and psychological inflexibility (AFQ-Y), which was added to the equation on step 2 (Table 3). The results indicated that other psychological constructs accounted for between 11 and 31% of the variance in psychopathological symptom scores (step 1). Most importantly, in the case of the YSR scales anxiety problems, affective problems, and oppositional/conduct problems (but not for CSI somatization), psychological inflexibility was found to make a significant, positive contribution when entering this variable on step 2, accounting for an extra 2 to 5% of the variance in these types of psychopathological symptoms. Other variables making consistent contributions to symptom scores were self-worth (YSR anxiety and affective problems) and self-compassion (YSR affective problems). In all these cases, beta weights were negative, indicating that lower self-worth and self-compassion scores were associated with higher symptom levels.

**Table 3:** Results of the regression analyses (standard beta weights are shown) conducted in sample 1 in which various types of psychopathological symptoms were predicted from various psychological constructs (entered on step 1) and psychological inflexibility (entered on step 2)

	CSI somatization		YSR anxiety problems		YSR affective problems		YSR oppositional/conduct problems	
	Step 1	Step 2	Step 1	Step 2	Step 1	Step 2	Step 1	Step 2
CAMM mindfulness	.17	.14	.12	.07	.23*	.17*	.21*	.17
WBSI thought suppression	.22*	.17	.25*	.16	.19*	.06	.01	−.07
S-SCS-A self-compassion	−.09	−.08	−.05	−.03	−.18*	−.15*	−.12	−.10
SPPC self-worth	−.12	−.10	−.25*	−.20*	−.21*	−.15*	−.14	−.10
AFQ-Y psychological inflexibility		.12		.21*		.30**		.20*
*F*	11.45**	1.84	15.14**	5.97*	21.85**	14.09**	5.93**	4.07*
(∆) *R* ^2^	.19	.01	.23	.02	.31	.05	.11	.02

*N* = 184. All regression analyses were corrected for gender by entering this variable on step 0

*AFQ-Y* Avoidance and Fusion Questionnaire for Youth, *CAMM* Child and Adolescent Mindfulness Measure, *WBSI* White Bear Suppression Inventory, *S-SCS-A* Shortened Self-Compassion Scale for Adolescents, *SPPC* Self-Perception Profile for Children, *CSI* Children’s Somatization Inventory, *YSR* Youth Self-Report

*p < .05, **p < .001

### Sample 2

#### General Findings

The reliability of the AFQ-Y was again satisfactory, with a Cronbach’s alpha of 0.89, and the internal consistency coefficients of the other scales were also good (range of alphas between 0.81 and 0.93; see Table 4). In sample 2, significant gender differences were found for psychological inflexibility [*t*(155) = 2.89, *p* < .01] and almost all other variables (all *t*s ≥ 2.32, *p*s < .05): girls scored higher on psychological inflexibility, thought suppression (WBSI), and symptoms of anxiety (both YAM-5 and RCADS) and depression (RCADS), whereas boys rated themselves as higher on mindfulness (CAMM), self-worth (SPPC), self-efficacy (SEQ-C), and symptoms of aggression (CRA).

**Table 4:** Descriptive statistics (means, standard deviations, gender differences, and internal consistency coefficients) for various measures obtained in sample 2

	Total sample (*N* = 157)	Boys (*n* = 50)	Girls (*n* = 107)	Cronbach’s α
AFQ-Y psychological inflexibility	18.00 (8.63)	15.16 (7.19)_a_	19.33 (8.95)_b_	0.89
CAMM mindfulness	26.31 (6.32)	29.50 (5.55)_a_	24.83 (6.13)_b_	0.81
WBSI thought suppression	42.43 (12.42)	37.56 (11.56)_a_	44.71 (12.91)_b_	0.92
S-SCS-A self-compassion	26.61 (6.54)	27.34 (6.01)_a_	26.26 (6.77)_a_	0.87
SPPC self-worth	16.81 (3.52)	18.14 (2.79)_a_	16.20 (3.66)_b_	0.93
SEQ-C self-efficacy	79.70 (12.54)	84.54 (12.62)_a_	77.44 (11.90)_b_	0.89
YAM-5 anxiety	13.74 (9.78)	8.74 (7.21)_a_	16.07 (9.97)_b_	0.92
RCADS anxiety	10.90 (7.76)	7.24 (6.50)_a_	12.61 (7.74)_b_	0.90
RCADS depression	2.93 (2.68)	1.74 (1.58)_a_	3.49 (2.91)_b_	0.79
CRA aggression	34.83 (8.28)	37.04 (9.77)_a_	33.79 (7.31)_b_	0.87

Means not sharing similar subscripts differ at *p* < .05

*AFQ-Y* Avoidance and Fusion Questionnaire for Youth, *CAMM* Child and Adolescent Mindfulness Measure, *WBSI* White Bear Suppression Inventory, *S-SCS-A* Shortened Self-Compassion Scale for Adolescents, *SPPC* Self-Perception Profile for Children, *SEQ-C* Self-Efficacy Questionnaire for Children, *YAM-5* Youth Anxiety Measure for DSM-5, *RCADS* Revised Child Anxiety and Depression Scale

#### Relations Between Psychological Inflexibility and Other Psychological Constructs/Symptoms

The correlations between psychological inflexibility and other constructs/symptoms showed the predicted pattern (Table 5). That is, the AFQ-Y was positively correlated with the WBSI (*r* = .58) and all symptom measures (YAM-5, RCADS, and CRA; *r*s between .19 and .58), whereas negative correlations were found with the CAMM (*r* = −.63), S-SCS-A (*r* = −.24), SPPS (*r* = −.38), and SEQ-C (*r* = −.43). This means that higher levels of psychological inflexibility were associated with higher levels of thought suppression and psychopathological symptoms, but lower levels of mindfulness and self-related constructs. Tests for comparing correlated correlation coefficients again showed that psychological inflexibility as indexed by the AFQ-Y was more strongly related to internalizing (anxiety and depression; *r*s ranging between .49 and .58) than to externalizing symptoms (aggression; *r* = .19; all *z*s ≥ 3.11, *p* < .01).

**Table 5:** Correlations (corrected for gender) among AFQ-Y and other measures (sample 2)

	1	2	3	4	5	6	7	8	9
1. AFQ-Y psychological inflexibility									
2. CAMM mindfulness	−.63**								
3. WBSI thought suppression	.58**	−.66**							
4. S-SCS-A self-compassion	−.24*	−.04	−.11						
5. SPPC self-worth	−.38**	.24*	−.29**	.47**					
6. SEQ-C self-efficacy	−.43**	.44**	−.35**	.23*	.52**				
7. YAM-5 anxiety	.58**	−.57**	.52**	−.18*	−.42**	−.53**			
8. RCADS anxiety	.49**	−.49**	.45**	−.10	−.37**	−.53**	.86**		
9. RCADS depression	.55**	−.52**	.45**	−.30**	−.52**	−.52**	.71**	.64**	
10. CRA aggression	.19*	−.24*	.13	−.07	.02	−.21*	.11	.13	.24*

*N* = 157

*AFQ-Y* Avoidance and Fusion Questionnaire for Youth, *CAMM* Child and Adolescent Mindfulness Measure, *WBSI* White Bear Suppression Inventory, *S-SCS-A* Shortened Self-Compassion Scale for Adolescents, *SPPC* Self-Perception Profile for Children, *SEQ-C* Self-Efficacy Questionnaire for Children, *YAM-5* Youth Anxiety Measure for DSM-5, *RCADS* Revised Child Anxiety and Depression Scale

*p < .05, **p < .001

As in sample 1, other psychological constructs were also interrelated (Table 5). That is, CAMM mindfulness was negatively associated with WBSI thought suppression (*r* = −.66, *p* < .001) and positively with SPPC self-worth (*r* = .24, *p* < .01), and SEQ-C self-efficacy (*r* = .44, *p* < .001). Further, WBSI thought suppression was negatively linked to SPPC self-worth and SEQ-C self-efficacy (*r*s being −.29 and −.35, respectively, both *p*s < .001). Finally, SPPC self-worth was positively related to S-SCS-A self-compassion and SEQ-C self-efficacy (*r*s being .47 and .52, respectively, both *p*s < .001).

#### Unique Contribution of Psychological Inflexibility to Symptoms

As shown in Table 6, the hierarchical regression analyses indicated that other psychological constructs accounted for between 11 and 43% of the variance in psychopathological symptom scores (step 1). Further, the results revealed that psychological inflexibility made a significant, positive contribution to the internalizing symptoms of anxiety (YAM-5 and RCADS) and depression (RCADS), accounting for an extra 1 to 2% of the variance (step 2). Other variables making consistent contributions to symptoms were CAMM mindfulness (YAM-5 anxiety, RCADS depression), SEQ-C self-efficacy (YAM-5 anxiety, RCADS anxiety, RCADS depression, CRA aggression), and SPPC self-worth (RCADS depression, CRA aggression). In most cases, beta values were negative indicating that lower levels of mindfulness, self-efficacy, and self-worth were accompanied by higher symptom levels. However, in the regression analysis predicting CRA aggression, the beta value for the SPPC was positive, implying that (when controlling for all other constructs) higher levels of aggression were related to higher levels of self-worth.

**Table 6:** Results of the hierarchical regression analyses (standard beta weights are shown) conducted in sample 2 in which various types of psychopathological symptoms were predicted from various psychological constructs (entered on step 1) and psychological inflexibility (entered on step 2)

	YAM-5 anxiety		RCADS anxiety		RCADS depression		CRA aggression	
	Step 1	Step 2	Step 1	Step 2	Step 1	Step 2	Step 1	Step 2
CAMM mindfulness	−.30*	−.21*	−.21*	−.12	−.35**	−.27*	−.26*	−.22
WBSI thought suppression	.18*	.14	.16	.12	.07	.03	−.06	−.08
S-SCS-A self-compassion	−.04	−.00	.04	.07	−.13*	−.10	−.15	−.13
SPPC self-worth	−.15*	−.13	−.12	−.10	−.27*	−.25*	.24*	.25*
SEQ-C self-efficacy	−.23*	−.22*	−.32**	−.31**	−.17*	−.16*	−.22*	−.21*
AFQ-Y psychological inflexibility		.21*		.18*		.17*		.08
*F*	26.72**	6.63*	18.55**	4.28*	27.22**	4.51*	3.77*	0.49
(∆) *R* ^2^	.41	.02	.34	.02	.43	.01	.11	.00

*Note*. *N* = 157. All regression analyses were corrected for gender by entering this variable on step 0

*AFQ-Y* Avoidance and Fusion Questionnaire for Youth, *CAMM* Child and Adolescent Mindfulness Measure, *WBSI* White Bear Suppression Inventory, *S-SCS-A* Shortened Self-Compassion Scale for Adolescents, *SPPC* Self-Perception Profile for Children, *SEQ-C* Self-Efficacy Questionnaire for Children, *YAM-5* Youth Anxiety Measure for DSM-5, *RCADS* Revised Child Anxiety and Depression Scale

*p < .05, **p < .001

## Discussion

The present study further examined the construct of psychological inflexibility in youths. Two independent samples of adolescents were asked to complete the AFQ-Y as well as a set of scales measuring other psychological concepts and psychopathological symptoms. First of all, it was found that in both samples, AFQ-Y scores correlated negatively with CAMM mindfulness and positively with WBSI thought suppression (see also Greco et al. 2008). This means that higher levels of psychological inflexibility are associated with a decreased tendency to be aware of and attend to internal and external events occurring in the present moment (e.g., Hanh 1976) and a stronger inclination to experience and suppress unwanted, intrusive thoughts (Wegner 1989). Factually, these results confirm that psychological inflexibility is characterized by both cognitive fusion (which is antithetical to mindfulness) and experiential avoidance (which is related to thought suppression; Kashdan and Rottenberg 2010), and thus can be best taken as support for the convergent validity of the AFQ-Y.

More importantly, findings indicated that the AFQ-Y was negatively linked to self-related concepts such as self-compassion, self-worth, and self-efficacy. Thus, higher levels of psychological inflexibility were accompanied by lower levels of (a) having compassion with oneself in times of adversity (Neff 2003a), (b) feeling oneself valuable and respectable as a person (Rosenberg 1979), and (c) one’s perceived ability to perform the required behaviors to achieve desired outcomes (Bandura 1977). These relations make sense as it is easy to see how cognitive fusion and experiential avoidance interfere with these adaptive features of the self. That is, self-compassion and self-worth are easily undermined when one’s thoughts are fused with self-criticism (Gohar et al. 2016; Hayes et al. 1999), and self-efficacy is crippled when a person is predominantly avoidant and lacks the flexibility to cope effectively with changing circumstances (Celikkaleli 2014).

Finally, the results show that psychological inflexibility in youths is positively associated with psychopathological symptoms. Even when controlling for the overlap with other psychological constructs, this variable still made a significant contribution in six out of eight regression analyses (samples 1 and 2 combined) predicting mental health symptoms. These findings provide evidence for the idea that psychological inflexibility is an important feature of a broad range of mental health problems (Kashdan and Rottenberg 2010) and that this is also the case in youth samples (Greco et al. 2008; Feinstein et al. 2011; Fergus et al. 2012; Livheim et al. 2016; Paulus et al. 2016; Simon and Verboon 2016; Valdivia-Salas et al. in press; Venta et al. 2012). Our data indicate that AFQ-Y scores were more substantially correlated with anxiety and depression than with oppositional/conduct problems and aggression, on the basis of which one might conclude that psychological inflexibility is more relevant for internalizing than for externalizing problems. However, it is also possible that these differential relationships were—at least in part—an artifact of the method used in this study. That is, unlike externalizing problems, internalizing symptoms are best assessed by means of self-report scales (De Los Reyes et al. 2015), which also happens to be the most optimal assessment method in case of the covert cognitive processes that characterize psychological inflexibility.

There were a number of additional findings that deserve brief discussion. First, as in previous studies (Greco et al. 2008; Livheim et al. 2016), the AFQ-Y proved to be a reliable index of psychological inflexibility in adolescents aged between 12 and 18 years. Second, gender effects on psychological inflexibility, and in its wake mindfulness and thought suppression, were not substantial: boys scored somewhat lower on the AFQ-Y and WBSI thought suppression but higher on CAMM mindfulness than did girls, but these differences were only significant in sample 2. This is in line with previous research: some studies did not show gender effects for psychological inflexibility, mindfulness, and thought suppression (e.g., Greco et al. 2011; Paulus et al. 2016), while other investigations documented small but statistically significant differences between boys and girls (Greco et al. 2008; Wegner and Zanakos 1994). Gender differences for other variables showed the expected pattern. That is, boys displayed high levels of self-worth (cf. Muris et al. 2003) and self-efficacy (cf. Muris 2001) as compared to girls. Further, girls reported higher levels of internalizing symptoms, whereas boys exhibited higher levels of externalizing problems (cf. Achenbach 2009). Third and finally, relations between psychological constructs and psychopathological symptoms were mostly in the expected direction. That is, mindfulness, self-compassion, self-worth, and self-efficacy were negatively associated with symptom levels (cf. Brown and Ryan 2003; MacBeth and Gumley 2012; Muris et al. 2003; Muris et al. 2011), whereas thought suppression was positively linked to problems (Magee et al. 2012). However, there was one noteworthy exception to this rule: in the regression analysis predicting symptoms of aggression from all psychological constructs, SPCC self-worth appeared to make a *positive* contribution. This result is in keeping with the notion that aggression is not necessarily associated with low levels of self-worth, but most commonly emerges in individuals with high levels of self-esteem when their highly favorable view of the self is disputed by some person or circumstance (Baumeister et al. 1996).

The present study suffers from various limitations. To begin with, this study only relied on youths’ self-report. The inclusion of other informants (e.g., parents) would have provided additional information and hence even more valuable insights in the psychological and psychopathological correlates of psychological inflexibility in young people. Another shortcoming pertains to the fact that this study was cross-sectional in nature, which means that no conclusions can be drawn on cause-effect relationships. For example, although we assume that psychological inflexibility is an antecedent of psychopathology, on the basis of the current data the possibility cannot be ruled out that psychological inflexibility is merely a consequence of mental health problems. Prospective studies are needed to explore its predictive role in the etiology and maintenance of internalizing and externalizing symptoms in youths, and it is good to see that such research is starting to emerge in the literature (Ciarrochi et al. 2011; Valdivia-Salas et al. in press). Further, the present investigation was conducted in a nonclinical sample of adolescents. Given the presumed relevance of psychological inflexibility for our understanding of mental health problems, it is particularly important to also conduct similar studies in clinically referred populations. Finally, for sample 2, the percentage of youths participating in the study was quite low, which of course questions the generalizability of findings. However, it was reassuring to note that the results obtained in sample 2 mimicked those documented for sample 1, in which the response rate was much higher.

In spite of these shortcomings, the current data indicate that psychological inflexibility is an important correlate of psychopathology in youths. This result paves the way for at least two lines of future research. The first line is concerned with the development of psychological inflexibility in young people, and thus pertains to the question why some children largely lack the ability to respond appropriately to environmental demands and internal experiences in the service of their goals. Tentative evidence suggests that parental levels of inflexibility and negative rearing behaviors (e.g., rejection, harsh discipline) are involved (Brassell et al. 2016; Williams et al. 2012), and of course this knowledge can be used to develop prevention programs. The second line of investigation pertains to the development and evaluation of treatment programs for children and adolescents with evident mental health problems. Specific interventions that target youths’ inflexibility by promoting (aspects of) psychological flexibility, such as acceptance and commitment therapy and mindfulness training, are increasingly evaluated (e.g., Livheim et al. 2015; Singh et al. 2007; Woidneck et al. 2014), and more of such studies are needed to further establish the empirical status of this new generation of behavioral therapies in youths. The AFQ-Y is recommended as an important measure that can be used in both lines of research: the present study further underlines that this scale provides a reliable and valid index of psychological inflexibility in young people.
